# Targeting the fundamentals for tremors: the frequency and amplitude coding in essential tremor

**DOI:** 10.1186/s12929-024-01112-8

**Published:** 2025-02-10

**Authors:** Ming-Kai Pan

**Affiliations:** 1 Department and Graduate Institute of Pharmacology, National Taiwan University College of Medicine, No. 1, Sec. 1, Ren-Ai Road, Taipei, 100 Taiwan; 2 Molecular Imaging Center, National Taiwan University, Taipei, Taiwan; 3 Institute of Biomedical Sciences, Academia Sinica, Taipei, Taiwan; 4 Cerebellar Research Center, National Taiwan University Hospital, Yun-Lin Branch, Yun-Lin, Taiwan; 5 Department of Medical Research, National Taiwan University Hospital, Taipei, Taiwan

**Keywords:** Essential tremor, Tremor, Amplitude, Frequency, Motor kinematics, Motor control, Cerebellum, Neuronal coding, Oscillations, Electroencephalogram

## Abstract

Essential tremor (ET) is one of the most common movement disorders with heterogeneous pathogenesis involving both genetic and environmental factors, which often results in variable therapeutic outcomes. Despite the diverse etiology, ET is defined by a core symptom of action tremor, an involuntary rhythmic movement that can be mathematically characterized by two parameters: tremor frequency and tremor amplitude. Recent advances in neural dynamics and clinical electrophysiology have provided valuable insights to explain how tremor frequency and amplitude are generated within the central nervous system. This review summarizes both animal and clinical evidence, encompassing the kinematic features of tremors, circuitry dynamics, and the neuronal coding mechanisms for the two parameters. Neural population coding within the olivocerebellum is implicated in determining tremor frequency, while the cerebellar circuitry synchrony and cerebellar-thalamo-cortical interactions play key roles in regulating tremor amplitude. Novel therapeutic strategies aimed at tuning tremor frequency and amplitude are also discussed. These neural dynamic approaches target the conserved mechanisms across ET patients with varying etiologies, offering the potential to develop universally effective therapies for ET.

## Introduction

**Fig. 1:**
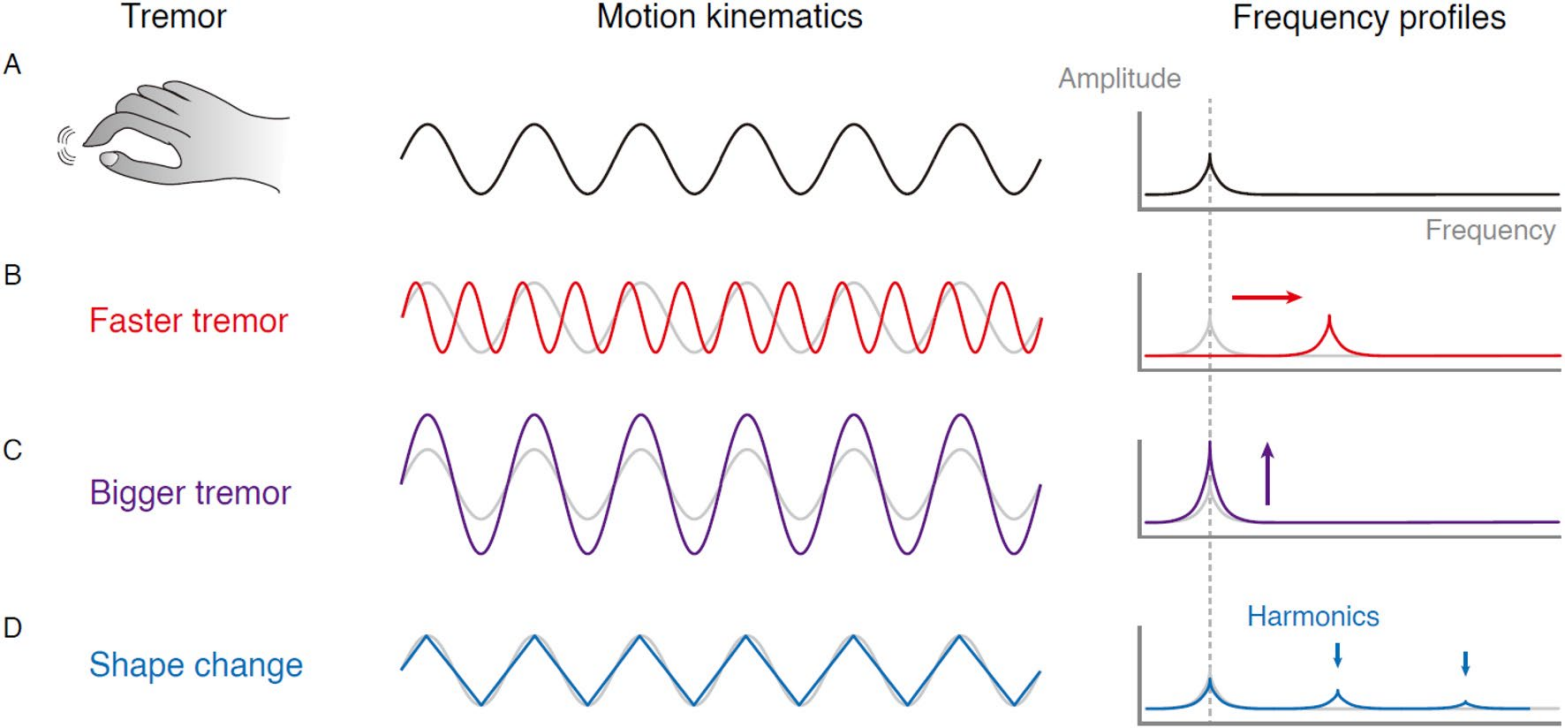
Description of tremor kinematics from the frequency domain. **A** Kinematics of tremors and corresponding frequency profiles. **B**–**D** Kinematic changes and corresponding effects in the frequency domain. Faster tremors lead to increased tremor frequency. Bigger tremors lead to increased tremor amplitude. Changing the shapes of the tremors leads to different profiles in the harmonic frequencies

Tremor is defined as an involuntary rhythmic movement with a fixed frequency. The mathematical characterization of tremors requires two key parameters: tremor frequency and tremor amplitude. Changes in tremor characteristics can be accurately described using these parameters (Fig. 1). Therefore, two functional networks are critical for understanding tremor generation: a rhythm-generating network and an amplitude-regulating network. A rhythm-generating network produces periodic muscle contractions by ensembles of neuronal oscillations in the peripheral or central nervous system. An amplitude-regulating network controls the amount of simultaneous muscle fiber contraction, related to the synchronized activation of spinal motor neurons. A tremor syndrome is highly correlated with its unique amplitude- and frequency-generating mechanisms. In physiological tremor, the frequency is related to the mechanical properties of the musculoskeletal system, and it can be modulated by altering the mass attached to the tremulous limb [1–3]. The amplitude of physiological tremor is influenced by beta-adrenergic receptors on skeletal muscle [4] and the synchronization of spinal motor outputs [5], explaining why stress increases tremor amplitude.

For all disabling tremor syndromes, essential tremor (ET) is the most prevalent, affecting approximately 4% of adults and 20% of elderly populations [6–9]. The core feature of ET is action tremor, an involuntary rhythmic movement that occurs during limb posturing (postural tremor) or movement (kinetic tremor) [10]. Anatomically, the cerebello-thalamo-cortical loop is predominantly involved in ET [11]. Strokes in the primary motor cortex, pontine nuclei, cerebellum, dentate nucleus, posterior part of the ventrolateral nucleus, and ventral intermediate nucleus (VIM) of the thalamus can lead to the cessation of tremors in ET patients [12–16]. Functional magnetic resonance imaging (fMRI) studies have provided additional evidence of activity and circuitry connectivity within these brain regions [12, 17–30]. The involved brain structures are part of the motor network, offering a foundation for further investigating the coding mechanisms for tremor frequencies and amplitudes.

In this review, we focus on the circuitry and neuronal mechanisms involved in the formation of tremor frequencies and the regulation of tremor amplitudes in ET.

## Main text

### Methods

We conducted a literature search on PubMed using the following keyword combinations: “tremor frequency phase essential tremor” (87 results), “tremor frequency modulation essential tremor” (67 results), and “tremor amplitude modulation essential tremor” (45 results). Each manuscript was reviewed, and only directly relevant evidence was included. Additional publications were incorporated as needed to connect and contextualize the segregated evidence.

## Results

### Kinematic features of ET

Understanding the neuronal coding mechanism for tremors requires detailed knowledge of the kinematic properties, particularly the temporal evolution of frequency and amplitude at sub-second resolution. ET patients have action tremors at the frequency range of 4–12 Hz [11]. Within individual patients, tremor frequency remains highly stable over extended periods, while tremor amplitude can fluctuate significantly over short intervals [31]. Detailed muscle kinematics reveal that the phase relationship between antagonistic muscle pairs is stable within the same posture but varies significantly between different postures [32]. Finger tapping in one limb can induce a shift in tremor frequency in the contralateral tremulous limb, suggesting an interaction between a self-controlled central oscillator and a tremor-related pathological oscillator [33]. The kinematic features of tremors in ET differ from those observed in parkinsonian rest tremors [34, 35] and dystonic tremors [36]. These differences can be distinguished using the tremor stability index [35] and other machine learning-based classifiers [34].

### Mapping neural circuitry for tremor frequency generation

#### Central origin of oscillatory circuitry in ET

Tremors can be generated from the peripheral muscular-skeletal system or oscillators in the central nervous system (CNS). In tremors with a peripheral origin, increasing the limb’s loading weight, which raises the inertia of the musculoskeletal system, typically reduces tremor frequency [37–39]. However, the tremor frequency in ET remains unchanged with weight-loading, suggesting that the peripheral mechanical system is not the source of the tremor rhythm or related oscillations [2, 3, 39]. Submotor-threshold peripheral stimulation, which activates intra-muscular sensory afferents, does not alleviate tremors or modulate tremor frequency, despite the wide ranges of stimulating frequencies and intensities [40].

#### Human evidence of frequency regulatory circuitry in ET

The 4–12 Hz tremors in ET indicate a CNS mechanism generating the same frequency-dependent oscillations. These sub-second temporal changes occur faster than the acquisition limits of functional magnetic resonance imaging (fMRI). Electrophysiological tools, such as magnetoencephalography (MEG) and electroencephalography (EEG), are required to capture these fast neural dynamics. MEG studies on ET postural tremors reveal frequency-dependent involvement of the premotor cortex, primary motor cortex, cerebellum, brainstem, and thalamus [41, 42]. Muscle-EEG coherence analyses also reveal frequency-dependent contributions from the primary motor cortex [43, 44]. Direct cerebellar EEG recordings have shown tremor-related cerebellar oscillations in ET patients but not in healthy subjects [45–47]. Intraoperative recordings of the cerebellar thalamus (ventral intermediate nucleus, or VIM) also reveal thalamic-muscle coherence during tremor but not at rest [48, 49].

Interventional tools, such as deep brain stimulation (DBS), transcranial magnetic stimulation (TMS), and transcranial alternating current stimulation (tACS), have been used to investigate whether tremor-related circuitry oscillations require the reciprocal interaction of the entire circuit or originate from a pacemaker structure or subcircuit. TMS applied to the primary motor cortex can “reset” tremors by replacing the current phases and timing of rhythmic muscular contractions with new onsets and phases after the TMS pulses [50–52]. The efficacy of tremor resetting correlates with the silent period following motor-evoked potentials [50], suggesting a potential contribution from cortico-projecting remote structures. tACS over the primary motor cortex can entrain postural, but not kinetic, tremors [53]. tACS over the cerebellum has shown more reliable entrainment effects on both postural and kinetic tremors [45, 53]. The dominant role of the cerebellum is further supported by intraoperative recordings of the cerebellar thalamus (VIM). Thalamic local field potential (LFP) oscillations typically precede the onset of neuronal burst activities, suggesting that thalamic neuronal activities may be entrained by periodic afferent inputs from the cerebellum [54]. A decisive evidence comes from simultaneous cerebellar EEG recordings during the on–off switches of thalamic DBS in ET patients [45]. Frequency-dependent oscillations persist regardless of DBS-on or DBS-off states, suggesting a cerebellum-to-thalamic information flow with potential frequency-forming subcircuits at the cerebellar level. Consistently, thalamic DBS suppresses tremor amplitudes but does not alter the tremor frequencies [45, 55].

In summary, current clinical evidence suggests a cerebellar-to-thalamic information flow for tremor-related circuitry oscillations, with potential frequency-forming subcircuits from the cerebellar parts.

#### Evidence of frequency-forming circuits in animal models

Animal studies offer direct interventional evidence from brain regions not yet accessible in clinical settings. Oscillations at the tremor frequency have been detected through LFP recordings of the cerebello-thalamo-cortical circuits in *Grid2*^*dupE3*^ tremor mice [45, 47], a tremor mouse model driven by ET cerebellar pathology with GluRδ2 loss and climbing fiber (CF) overgrowth. The tremor-frequency-matched oscillations observed in the primary motor cortex, thalamus, and cerebellar cortex align with clinical findings and are further supported by LFP recordings in the inferior olive (IO) and deep cerebellar nucleus (DCN). This finding supports the hypothesis that frequency-dependent oscillations exist in the entire tremor circuitry. DBS-mimicking thalamic blocking by lidocaine successfully suppresses mouse tremors and oscillations in the primary motor cortex, but the cerebellar oscillations remain [45]. Direct silencing of cerebellar neurons with lidocaine [45] or optogenetic silencing of cerebellar Purkinje cells (PCs) [45, 47] successfully halts both tremors and frequency-dependent oscillations across the entire circuit. These findings support clinical observations that tremor-related circuitry oscillations originate in the cerebellum and propagate to the motor cortex via the thalamus.

Animal studies further clarify that the olivocerebellar circuit—a closed-loop system involving IOs, cerebellar PCs, and DCNs, generates frequency-dependent oscillations for tremors. Silencing IO neurons or disrupting their synaptic vesicle release at CF to PC synapses suppresses tremors and related cerebellar oscillations [45, 47]. Harmaline-induced rhythmicity and synchronicity of IO neurons can generate acute tremors in rodents, cats, and primates [56–60]. Disrupting PC axonal outputs can suppress tremor-related circuitry oscillations and tremors in both *Grid2*^*dupE3*^ and harmaline-induced tremor mouse models [45, 47, 56]. Silencing any component of the olivocerebellar circuit disrupts the entire circuitry oscillations and eliminates tremors in mice [45]. Detailed analysis of circuitry oscillations has shown that the oscillatory activities in the IOs, cerebellar cortex, and DCNs are highly coherent at the tremor frequency [45].

In summary, animal studies suggest that tremor frequency-related circuitry oscillations originate from the olivocerebellum, propagate to the motor cortex via the thalamus, and generate tremors. These findings align with clinical observations, where thalamic DBS does not halt cerebellar oscillations, but cerebellar interventions such as TMS or tACS show frequency modulation effects for tremors in ET patients.

### Cellular mechanisms for tremor frequency formation

#### Neuron populational activity decides tremor frequency

The above review provides evidence of oscillation-forming circuitry for tremor frequency, but a key question remains unanswered: if an ET patient has a 6-Hz action tremor, how is this “6” being computed in the tremor circuit? Addressing this question requires mechanisms with numerical precision and mathematical validation. For instance, 7-Hz olivocerebellar oscillations cannot account for a 6-Hz tremor.

Harmaline induces 6–12 Hz burst firings in IO neurons, compatible with the frequency of harmaline-induced tremors [61, 62]. The burst activity in these neurons leads to comparable 6–12 Hz complex spikes in PCs [62, 63]. Modern recording techniques using electrode arrays in awake-behaving mice allow for the evaluation of neuronal activities in large populations with simultaneously recorded motor kinematics. While the LFP frequency in the olivocerebellum matches the tremor frequency, the individual neuronal firing rates are not [45]. The neuronal firing rates across IO, DCN, and PCs show poor correlation with tremor frequency, and the firing probability is poorly correlated with tremor phases at the single-cell level [45]. However, when examining multiple neurons within the same location (e.g. in the IO), the summated firing probability at the population level begins to exhibit periodicity, converging on the LFP frequency and matching the tremor frequency [45]. This phenomenon is conserved across the IO, DCN, and PCs [45]. Thus, the olivocerebellum uses populational firing probability to compute the final frequency outcome, a biological strategy to approach the expected frequency value through simultaneous sampling by a large population of neurons with intrinsic noise. The artificial creation of periodic population activity via optogenetic stimulation can generate cerebellar oscillations and tremors at the stimulating frequency [45, 47, 64]. In ET patients, in-phase tACS over the cerebellum can produce more sinusoidal postural tremors with enhanced frequency stability, likely due to the consolidation of population firing probability at the tremor frequency. Consistently, out-of-phase tACS may disrupt cerebellar population coding, leading to reduced tremor frequency stability [45].

Neuronal population coding also reveals circuitry interactions. IO oscillations exhibit a 120-degree phase lead over oscillations in the cerebellar cortex, which is in antiphase (180 degrees) with oscillations in the DCN [45]. This phase relationship is supported by the glutamatergic long projections from the IO to PCs and the GABAergic outputs from PCs to the DCN. Moreover, delivering a 13-Hz optogenetic stimulation to any of the three locations within the olivocerebellum in *Grid2*^*dupE3*^ mice with a 20-Hz tremor can shift the tremor to 13 Hz by replacing the 20-Hz oscillations with 13-Hz oscillations across the entire olivocerebellum [45]. This finding indicates that frequency-dependent cerebellar oscillations require coherent circuitry interaction across all three structures of the olivocerebellum.

**Fig. 2:**
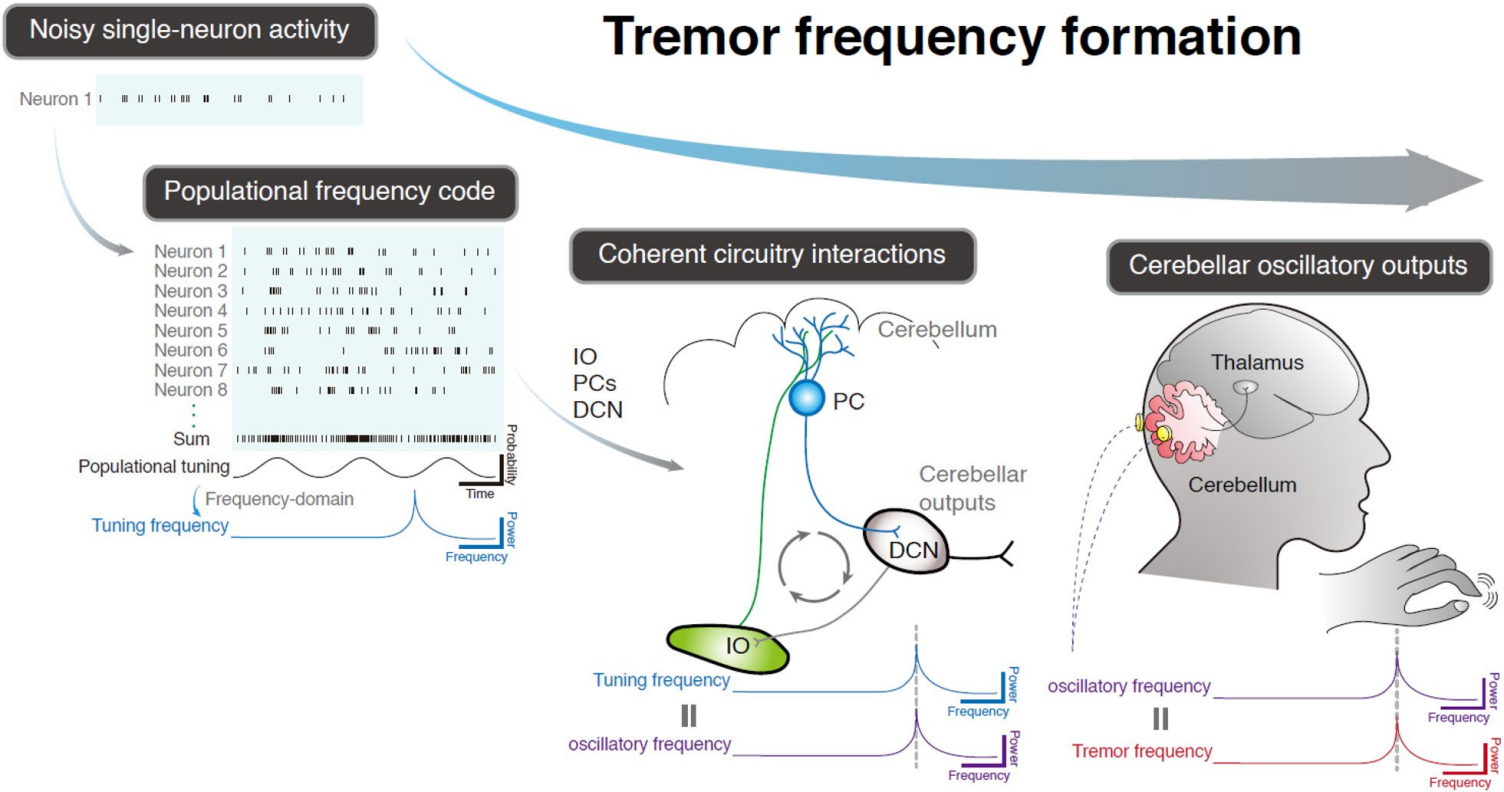
Mechanisms for tremor frequency formation. Neurons involved in tremor frequency formation exhibit unstable firing probability at the single cell level, but the combined firing probabilities of multiple neurons converge to a stable periodicity, resulting in a tuning frequency at the populational level. This populational coding mechanism is consistently presented across structures of the olivocerebellum, including IO neurons, PCs and DCN neurons, leading to stable circuitry oscillations at the tuning frequency. The circuitry oscillations cause tremors and can be picked up by cerebellar EEG, showing that the oscillatory frequency matches the tremor frequency

In summary, the exact value of tremor frequency is computed by the population activity of neuronal firing probabilities within the olivocerebellum. The resulting oscillatory frequency is a product of coherent circuitry interaction, rather than being driven by a single dominant pace-making structure. The frequency formatting mechanisms from cellular to circuitry levels are summarized in Fig. 2 and Table 1.

**Table 1:** Mechanisms related to tremor frequency formation

Mechanism	Species	Methods	Description	References
Cellular				
IO bursting	Cats	Intra- and extracellular recording	IO bursting frequencies fall into harmaline-induced tremor frequencies	[61, 62]
PC complex spikes	Rodents	Extracellular recording and microdialysis	Frequencies of PC complex spikes fall into harmaline-induced tremor frequencies	[62, 63]
Population frequency code	Rodents	Extracellular recording	The neuronal firing probability converges to the tremor frequency at the populational level among IO neurons, PCs or DCN neurons	[45]
	Rodents	Optogenetics	Rhythmic optogenetic stimulation at IO neurons, PCs or DCN neurons leads to tremor at the stimulating frequency	[45, 47, 64]
Structure				
Olivocerebellar synchony	Rodents	Extracellular, LFP recordings and optogenetics	The IO, PCs and DCN of the olivocerebellum oscillates coherently to determine tremor frequency	[45]
Cerebellar oscillations	Rodents and patients	LFP (rodents), EEG (patients)	Cerebellar oscillatory frequency matches tremor frequency	[45–47]
Cerebellar frequency entrainment	Patients	tACS	Frequency-dependent cerebellar tACS bidirectionally modulates tremor frequency stability	[45]
Thalamic oscillations	Patients	Intra-operative recording	The thalamus and muscles show coherence during tremor	[48, 49]
Cortical tremor entrainment	Patients	tACS	tACS entrains posture but not kinetic tremor	[53]
Cortical tremor resetting	Patients	TMS	TMS at primary motor cortex resets tremor and correlated with the silent period	[50–52, 54]
Circuit				
Cerebello-thalamo-cortical propagation	Rodents and patients	LFP (rodents), EEG (patients)	Cerebellar oscillations propagate via the cerebello-thalamo-cortical pathway and are suppressible by thalamic DBS	[45, 47]
Circuitry oscillations	Patients	MEG	Frequency-dependent oscillations in premotor cortex, primary motor cortex, the cerebellum, brainstem and thalamus	[41, 42]

### Underlying ion channel properties and pharmacology targeting on frequency generation

The frequency-generating properties of the olivocerebellum are primarily supported by the automaticity and intrinsic firing properties of IO neurons, PCs, and DCN neurons. These neurons can generate periodic firing of action potentials without synaptic inputs. The intrinsic pace-making features, when exposed to the circuitry augmentations via neuronal synchrony (see next section), lead to excessive circuitry oscillations and thus tremors. Among the olivocerebellar neurons, hyperpolarization-activated cyclic nucleotide-gated (HCN) channels and T-type calcium channels are key players in generating intrinsic oscillatory properties.

In IO neurons, HCN channels act as pacemakers and frequency filters, facilitating rhythmic activity within the 2–10 Hz range [65]. T-type calcium channels collaborate with HCN channels, amplifying subthreshold neuronal oscillations into rhythmic action potentials and bursts [65]. These channels are also essential for the generation of harmaline-induced tremors [60]. In PCs, HCN channels contribute to self-pacing, while T-type calcium channels support CF-dependent complex spikes, which play a significant role in tremor generation in mice [47, 56]. In ET patients, leaky ryanodine receptor type 1 in PCs causes periodic calcium fluctuations, contributing to tremor pathophysiology [66]. The rebound burst firings of DCN neurons, triggered by GABAergic activity from PC outputs [67], are supported by T-type calcium channels.

In terms of ET pharmacology, propranolol is one of the two FDA-approved medications and functions as an inhibitor of beta-adrenergic receptors. Interestingly, the conductance of HCN channels is regulated by beta-adrenergic receptors through cAMP pathways [68], potentially explaining the modulatory effects on cerebellar circuits in harmaline-induced tremor [69]. Beyond propranolol, T-type calcium channels are emerging as novel targets for ET treatment, with several pharmaceutical companies pursuing this approach [70–75].

In summary, the intrinsic firing properties of olivocerebellar neurons, driven by HCN and T-type calcium channels, are essential for the generation of tremor-related oscillations. These ion channels serve as key pharmacological targets in ET, with ongoing research focusing on their modulation to alleviate tremor symptoms.

### Circuitry contributions to tremor amplitudes

Besides frequency, amplitude is the other fundamental parameter for mathematically describing tremors. Many pathophysiological discoveries are associated with tremor amplitudes, which are directly linked to the severity scores for ET. Among the structures within the cortico-ponto-cerebellar-thalamo-cortical loop, the cerebellum is the most consistently involved structure [16, 76], and the reduction of its functional connectivity to sensorimotor cortices is correlated with tremor severity [12, 30].

It is worth noting that the tremor amplitudes are based on rhythmic movement generated by the frequency-determining mechanism discussed earlier, making them distinct from the amplitude-related pathophysiology observed in disorders like myoclonus, chorea, or ballism.

#### Amplitude modulators within the frequency-generating circuitry

Cerebellar EEG recordings in ET patients show a positive correlation between the strength of frequency-dependent cerebellar oscillations and tremor severity [46, 47, 77]. Since EEG records the spatial and temporal summations of neuronal activities, this correlation implies enhanced neuronal synchrony. The hypothesis of neuronal synchrony is supported by the CF lateral crossings on PC dendrites in ET cerebellar pathology [78], which can crosslink neighboring PCs and lead to excessive synchrony. The lateral crossing pathology also aligns with the pathophysiology of GluRδ2 loss and CF outgrowth in ET [47, 79], where GluRδ2 loss disrupts CF competition, leading to multiple CF innervations per PC [80]. PC axonal torpedoes are observed in ET, and the number of torpedoes is associated with the tremor severity [79]. Such PC swellings could increase axonal conduction fidelity, enhancing PC-to-DCN axonal transmission and temporal synchrony [81]. An increase in recurrent collateral axons of Purkinje cells (PCs) is also a consistent finding in ET [79, 82, 83]. These PC-to-PC collaterals exhibit a strong co-activating effect on connected PCs, thereby promoting PC synchrony [84].

In animal studies, GluRδ2-loss-related CF overgrowth is directly linked to increased cerebellar oscillations [47], echoing the pathology and cerebellar EEG findings for tremor amplitude modulation in ET patients. Rhythmic optogenetic stimulation in the DCN can induce mouse tremors at the illuminating frequency, and the tremor amplitudes are correlated with light intensity [64], directly controlling the number of neurons activated synchronously. Additionally, increased synchrony of IO neurons also modulates tremor amplitude, as shown by IO injections of picrotoxin, which augmented IO coupling and synchronized complex spikes in multiple PCs [85, 86]. Collectively, the frequency of the olivocerebellar oscillations regulates the tremor frequency, while the synchrony of neuronal activities within the IO, DCN, or PCs contributes to the frequency-dependent oscillatory strength and tremor amplitudes.

In contrast to the precise coding of tremor frequency by the olivocerebellum, cerebellar oscillatory strength, even when tightly regulated by optogenetic manipulation, cannot fully explain changes in tremor amplitudes, suggesting the presence of additional amplitude modulators.

#### Amplitude modulators outside of the frequency-generating circuitry

Cerebellar-thalamic functional connectivity is positively correlated with tremor severity [12]. The thalamus acts as a gatekeeper, gating the propagation of cerebellar oscillations to the primary motor cortex, thereby modulating tremor amplitudes. Intraoperative recordings show a strong correlation between thalamic neuronal inhibition and tremor suppression [87]. At therapeutic levels of thalamic DBS, thalamo-cortical evoked potentials are undetectable, suggesting that tremor suppression is due to thalamic silencing rather than activation [88]. DBS at the posterior subthalamic area (PSA), which contains cerebellar output tracts, has comparable efficacy as VIM DBS [89], if not better [90]. Magnetic resonance-guided focus ultrasound (MRgFus) also demonstrates significant tremor reduction by lesioning the cerebellar thalamus (VIM) [91] or cerebellothalamic tract [92]. In the harmaline rodent model, thalamic oscillations at tremor frequency are correlated with tremor amplitudes [93]. Collectively, these findings underscore the thalamus’s role as a gating resistor regulating tremor amplitudes.

Thalamo-cortical interactions further contribute to tremor amplitude modulation in ET. Reduced thalamo-cortical functional connectivity correlates with tremor severity [12, 30]. Thalamo-cortical interactions differ significantly between “tremor-on” and “tremor-off” states in ET patients undergoing DBS surgery, with modulation varying across frequency bands. Theta and beta (13–30 Hz) activity in M1 increases acutely after thalamotomy and the activity can be suppressed by posturing [94]. In the harmaline rat model, theta and high beta oscillations increase in both the cerebellar thalamus and M1, and thalamo-cortical coherence increases at the subharmonic frequency of harmaline-induced tremor [93, 95]. These findings indicate that thalamo-cortical interactions are critical in regulating tremor amplitudes, particularly in gating “tremor-on” and “tremor-off” states. Notably, the connectivity changes vary across frequency bands, leading to conflicting interpretations between fMRI and electrophysiological approaches.

#### Spinal and peripheral modulators for tremor amplitudes

The evidence regarding peripheral modulation of tremor amplitudes in ET patients is less clear. The cutaneous silent period, a spinal inhibitory reflex, is significantly prolonged in ET patients and can be partially corrected by propranolol administration [96]. H-reflex condition is also abnormal in ET patients and can be improved by botulinum toxin injection [97]. Stress and sympathomimetics can act on muscle spindles and synchronize motor outflows via spinal reflex modulation [5], which may explain the increased tremor amplitudes in ET patients under stress.

**Fig. 3:**
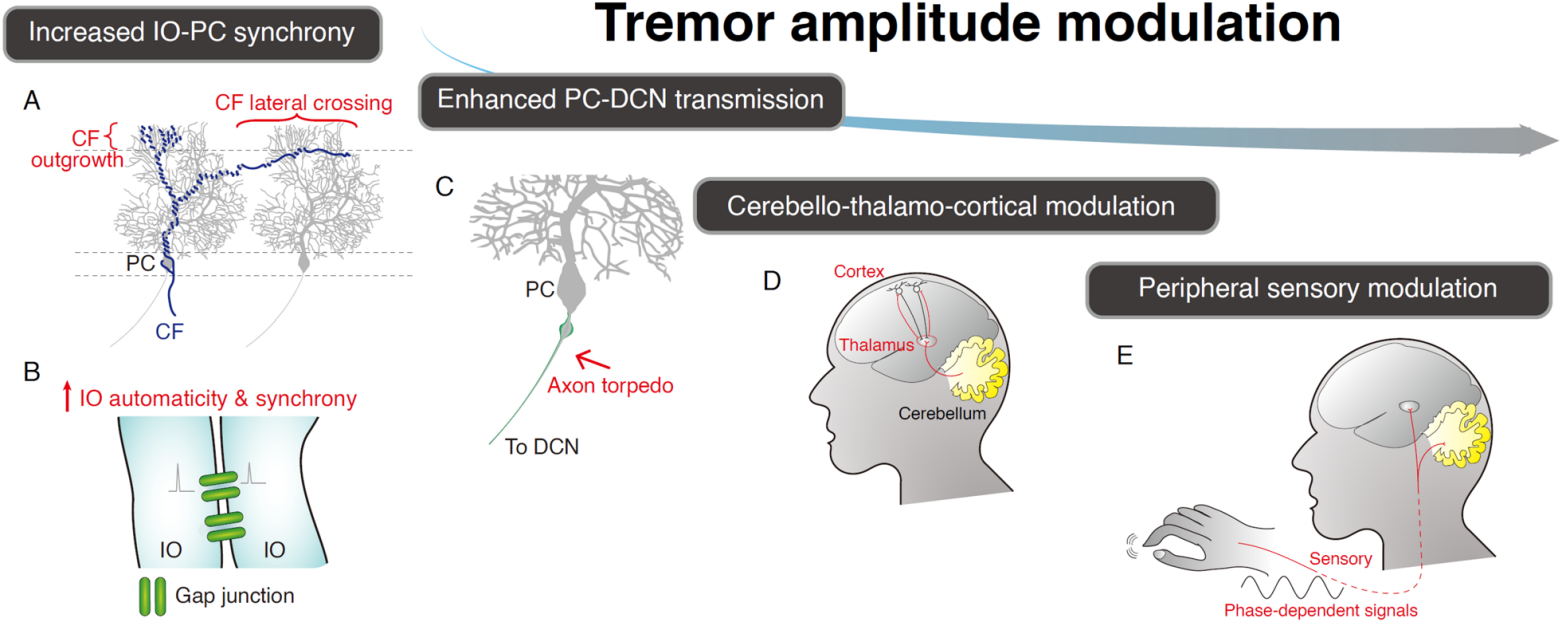
Mechanisms for tremor amplitude modulation. **A**, **B** Increased IO-PC synchrony. CF overgrowth, or enhanced IO automaticity and coupling, increases synchrony between IO neurons and PCs, as well as within the IO and among PCs. **C** Enhanced PC-to-DCN transmission. Axonal torpedoes in PCs enhance the transmission from PCs to DCN neurons. **D** Cerebello-thalamo-cortical modulation. The thalamus modulates tremor amplitudes by gating cerebellar-to-cortical transmission, as well as through thalamo-cortical interactions. **E** Peripheral sensory modulation. Phasic sensory inputs from peripheral nerves influence tremor amplitudes in a frequency-dependent manner

In summary, tremor amplitudes are directly linked to the cerebellar oscillatory strength at the tremor frequency. The thalamus plays a key role in gating oscillatory propagation from the cerebellum to the motor cortex, significantly contributing to amplitude modulation. Thalamo-cortical interactions are crucial in regulating tremor amplitudes, particularly in gating “tremor-on” and “tremor-off” states, with distinct effects across different frequency bands. Spinal reflex alterations are noted in ET patients, but there is not enough evidence to conclude their contributions to tremors. The amplitude modulatory mechanisms from cellular to circuitry levels are summarized in Fig. 3 and Table 2.

**Table 2:** Mechanisms related to tremor amplitude modulation

Mechanism	Species	Methods	Description	References
Cellular				
IO neuronal synchrony	Rodents	Pharmacology	Augmented gap-junction-mediated IO coupling enhances tremor amplitudes	[85, 86]
PC loss of GluRδ2 protein	Rodents and patients	Pathology	Reduction of GluRδ2 correlates with CF overgrowth and tremor severity	[47, 79]
PC axonal torpedoes	Patients	Pathology	Number of torpedoes correlates with tremor severity	[79]
PC recurrent collateral axons	Patients	Pathology	Increased PC-to-PC connections via recurrent collateral axons	[79, 82, 83]
CF overgrowth and lateral crossing	Rodents and patients	Pathology	Tremor severity is correlated with CF overgrowth to distal PC dendrites and lateral crossing to neighboring PCs	[47, 78, 79]
DCN neuronal synchrony	Rodents	Optogenetics	Light intensity of rhythmic illumination correlates with tremor intensity	[64]
Thalamic neuronal activity	Patients	Intra-operative recording	Thalamic neuronal inhibition is correlated with tremor suppression	[87, 88]
Structure				
Cerebellar oscillations	Patients	EEG	Frequency-dependent oscillatory strength is correlated with tremor severity	[46, 47, 77]
	Patients	tACS	Phase-dependent tACS over the cerebellum bidirectionally modulates tremor severity	[45, 113]
Thalamic silencing	Patients	DBS	Phase-dependent DBS silencing suppress tremors	[98, 99]
Thalamic oscillations	Rodents	LFP	Amplitudes of thalamic oscillations at tremor frequency are correlated with tremor severity	[93]
Cerebral oscillations	Rodents and patients	LFP (rodents) EEG (patients)	Reduction of theta and beta oscillations at the primary motor cortex is correlated with tremor severity	[94, 95]
Peripheral nerve inputs	Patients	Nerve stimulation	Out-of-phase nerve stimulation at tremor frequency suppresses tremor	[115–118]
Circuit				
Cerebellar-thalamic connectivity	Patients	fMRI	Cerebellar-thalamic functional connectivity is positively correlated with tremor severity	[12]
Thalamo-cortical connectivity	Patients	fMRI	Reduction of thalamocortical functional connectivity is correlated with tremor severity	[12, 30]
Thalamo-cortical connectivity	Patients	EEG	Thalamus-dependent M1 activity at theta and beta band are suppressed by tremors	[94]
Thalamo-cortical connectivity	Rodents	LFP	Thalamo-cortical coherence increased during harmaline-induced tremor	[93, 95]
Cerebellar-sensorimotor connectivity	Patients	fMRI	Reduction of functional connectivity between the cerebellum and sensorimotor cortex correlates with tremor severity	[12, 30]

### Therapies targeting the rhythmic nature of tremors

Therapies for ET have been extensively reviewed in previous literature. Here, we focus on new strategies targeting the rhythmic nature of tremors.

#### Phase-locked DBS

Conventional DBS applies fixed frequency and amplitude parameters. Leveraging the rhythmic nature of tremors, phase-locked DBS has been developed to calibrate DBS stimulation timing based on limb-tremor phases. Phase-locked DBS successfully modulates tremor amplitudes [98, 99], with amplitude-phase responses following the Wilson-Cowan model [100]. This approach potentially conserves battery life by reducing continuous stimulation. However, it remains unclear whether this DBS design can prevent the tolerance observed in long-term DBS therapies for ET [101–111].

#### Repetitive TMS (rTMS)

rTMS over the cerebellum aims to disrupt the function of the oscillatory generator, revealing a tremor-reduction effect. A single session of low-frequency (1 Hz) cerebellar rTMS causes transient tremor reduction, while a 5-day consecutive course has a prolonged effect lasting up to 3 weeks [112].

#### tACS

Based on the cerebellum's role in frequency-dependent oscillations, rhythmic tACS designs have been tested as a proof-of-concept tremor therapy. Dynamic phase-tracking tACS over the cerebellum shows bidirectional effects on amplitude modulation [113]. This technology dynamically tracks phases of posture tremors and applies cycling tACS with fixed phase lags between tACS currents and tremors. Each patient has a personalized phase range with tremor-suppressing effects due to varying nerve conduction times [113]. A different approach is to apply cerebellar tACS at the patient’s tremor frequency, which also generates reversible and bidirectional modulation to tremor frequencies and tremor amplitudes [45]. A tremor-frequency-disturbing tACS protocol, which disrupts frequency-dependent cerebellar oscillations with anti-phase tACS currents, suppresses tremor amplitudes in ET patients [45].

#### Peripheral nerve stimulation

Peripheral nerve stimulation does not modulate tremors unless designed to interact with the central frequency generator [40]. However, when phase-locked to tremors and aligned with tremor frequency, peripheral stimulation provides modulatory effects [114]. Tremor reduction occurs when peripheral stimulation is out-of-phase with the tremor [115–118]. Notably, sensory stimulation did not change the tremor frequency [40, 119]. Supramaximal nerve stimulation can at least partially reset tremors [120]. Sinusoidal external force applied to the wrist can entrain tremor frequency [121].

#### MRgFus

MRgFUS is a cutting-edge technology for tremor therapy that has been extensively reviewed [122–124]. This technique utilizes multiple ultrasound probes to converge mechanical energy onto a specific brain region, generating thermal lesions without opening the skull. The targeted brain region is first validated using magnetic resonance thermometry during a phase of reversible, non-lesioning heating, significantly enhancing the safety and precision of the procedure. For tremor therapy, MRgFUS targets the cerebellar thalamus (VIM) [91] or cerebellothalamic tract [92], aligning with the “quarantine effect” highlighted in this review. This effect involves disrupting tremor-related olivocerebellar oscillations, preventing their propagation to the motor cortex via the cerebellar-thalamo-cortical pathway.

In summary, therapies targeting the rhythmic nature of tremors, such as phase-locked DBS, rTMS, tACS, and peripheral nerve stimulation, offer promising avenues for tremor modulation in ET. These approaches leverage the underlying oscillatory mechanisms, with phase-locking and frequency alignment proving crucial for their effectiveness. Ongoing research is needed to refine these therapies and fully understand their long-term impact on tremor management.

## Discussion

While our understanding of these mechanisms has advanced, it also opens the door to new questions. We have elucidated the neuronal and circuitry mechanisms underlying tremor frequency generation in the olivocerebellum, but deeper inquiries remain. For instance, why does one patient exhibit a 6 Hz tremor while another has a 7 Hz tremor? What are the underlying mechanisms that determine the target frequency? Why do tremor frequencies decrease and amplitudes increase with aging or prolonged disease duration? Why is ET action-dependent? Addressing these questions will bring us closer to understanding the core pathophysiology of tremors and refining therapeutic approaches.

This review also underscores the importance of neural dynamics and their complementary role in therapeutic development. For example, propranolol, a common treatment for ET, modulates the pace-making properties of IO neurons, thereby altering circuitry oscillations. Although this therapeutic strategy targets pathological circuitry dynamics, it is important to recognize that the expression of beta-adrenergic receptors, the binding site of propranolol, is not different from those in healthy individuals. Consequently, the mechanism may not be detectable through genetic or molecular biology approaches. The integration of neural dynamic perspectives offers a fresh angle for uncovering pathophysiological mechanisms and designing novel therapies.

## Conclusions

Tremor frequency and amplitude are two key parameters for describing tremor kinematics. In this review, we summarized the current evidence on tremor frequency and amplitude coding in ET. The olivocerebellum plays a central role in the computation of tremor frequency and the generation of frequency-dependent oscillations. Tremor frequency arises from the integrated neuronal firing probabilities at the population level, which converge on a specific cerebellar oscillatory frequency. The strength of these oscillations, driven by the synchrony of olivocerebellar neurons, significantly influences tremor amplitude. The tremor amplitudes are further modulated by the cerebellar-thalamic and thalamo-cortical pathways. Novel therapeutic strategies based on the rhythmic nature of tremors show promising effects. ET is known as a disease with diverse etiology. Targeting the mechanisms of the core features across all ET patients provides a new perspective to identify conserved mechanisms and the potential for developing universal therapies.

## Data Availability

Not applicable.

## Abbreviations

CF: Climbing fiber
CNS: Central nervous system
DBS: Deep brain stimulation
DCN: Deep cerebellar nucleus
EEG: Electroencephalography
ET: Essential tremor
fMRI: Functional magnetic resonance imaging
IO: Inferior olive
LFP: Local field potential
MEG: Magnetoencephalography
MRgFUS: Magnetic resonance-guided focused ultrasound
PC: Purkinje cell
PSA: Posterior subthalamic area
rTMS: Repetitive transcranial magnetic stimulation
tACS: Transcranial alternating current stimulation
TMS: Transcranial magnetic stimulation
VIM: Ventral intermediate nucleus
